# PR3-ANCA-Associated Vasculitis in IgGκ MGUS: A Fatal Case of Rapidly Progressive Glomerulonephritis

**DOI:** 10.3390/jcm15072554

**Published:** 2026-03-27

**Authors:** Carlos Berrocal, Álvaro Arbeláez-Cortés, Alyi Arellano, Antonio Peña, H. A. Nati-Castillo, Nancy Mejia, Alice Gaibor-Pazmiño, Marlon Arias-Intriago, Juan S. Izquierdo-Condoy

**Affiliations:** 1 Departamento de Medicina Interna, Universidad del Valle, Cali 760032, Colombia; 2 Programa de Medicina, Facultad de Salud, Universidad Santiago de Cali, Cali 760045, Colombia; 3 One Health Research Group, Universidad de Las Américas, Quito 170125, Ecuador; 4 Pathology Department, SOLCA (Sociedad de Lucha Contra el Cáncer del Ecuador), Quito 170138, Ecuador

**Keywords:** rapidly progressive glomerulonephritis, ANCA-associated vasculitis, monoclonal gammopathy of undetermined significance, acute kidney injury, pauci-immune glomerulonephritis

## Abstract

**Background**: Rapidly progressive glomerulonephritis (RPGN) is a severe nephrological emergency, frequently secondary to anti-neutrophil cytoplasmic antibody (ANCA)-associated vasculitis. In older adults, the coexistence of comorbidities and monoclonal gammopathy of undetermined significance (MGUS) makes it difficult to distinguish between ANCA vasculitis and monoclonal gammopathy of renal significance (MGRS), which differ in prognosis and treatment. The coexistence of PR3-ANCA-associated vasculitis and MGUS is uncommon and sparsely documented. **Case Presentation:** A 72-year-old woman with hypertension and type 2 diabetes presented with acute deterioration and rapidly progressive renal failure, requiring hemodialysis. She had subnephrotic proteinuria, hematuria, and an active urinary sediment. The autoimmune workup showed ANCA negativity using immunofluorescence, but PR3-ANCA positivity using ELISA. Hematologic characterization documented an IgG kappa monoclonal spike; no bone lesions, amyloidosis, or criteria for multiple myeloma were found; and the patient was classified as MGUS. Renal biopsy revealed necrotizing extracapillary pauci-immune glomerulonephritis with cellular and fibrocellular crescents and no monoclonal deposits, consistent with PR3-ANCA vasculitis. Induction therapy with methylprednisolone pulses and oral prednisone was initiated; cyclophosphamide was not administered because of catheter-associated Staphylococcus aureus bacteremia and upper gastrointestinal bleeding complicated by disseminated intravascular coagulation. The patient died on day 25 due to infectious and hemorrhagic complications. **Conclusions**: This case provides additional documentation of an uncommon overlap between PR3-ANCA-associated vasculitis and MGUS in a Latin American patient and highlights the role of renal biopsy in distinguishing MGRS from pauci-immune vasculitis in the presence of paraproteinemia. It also underscores the need to tailor immunosuppression in frail older adults, balancing disease control against the risk of severe infection.

## 1. Introduction

Rapidly progressive glomerulonephritis (RPGN) is one of the most severe nephrological emergencies, characterized by an accelerated deterioration of renal function over days or weeks and histopathological findings of extracapillary proliferation with crescent formation in more than 50% of affected glomeruli [1]. Among its main etiologies are anti-neutrophil cytoplasmic antibody (ANCA)-associated vasculitis, which are responsible for a spectrum of pauci-immune systemic diseases involving several tissues, particularly the kidney, lung, and peripheral nervous system [2]. These entities require timely diagnosis and intensive immunosuppressive treatment, since the absence of rapid intervention may lead to end-stage kidney disease (ESKD) or even death, especially in older adults and in patients with severe or extrarenal forms [2,3].

In this age group, the coexistence of comorbidities such as diabetes, arterial hypertension, or monoclonal gammopathy of undetermined significance (MGUS) poses additional diagnostic and therapeutic challenges. In particular, distinguishing between ANCA vasculitis with renal involvement and monoclonal gammopathy of renal significance (MGRS) is crucial, since both entities may present with a nephritic or rapidly progressive syndrome but differ substantially in terms of prognosis and management: while ANCA-associated vasculitis requires intensive immunosuppression, MGRS is treated with therapies directed against the underlying B-cell or plasma-cell clone [4,5].

In this context, the coexistence of monoclonal gammopathy and ANCA-associated renal disease appears to be sparsely documented and possibly underrecognized rather than unequivocally rare [4,6,7,8,9], particularly when monoclonal studies are not performed systematically [10]. Nevertheless, biopsy-proven PR3-ANCA pauci-immune crescentic glomerulonephritis occurring in a patient with MGUS remains uncommon in the published literature, and appears to be particularly seldom reported in Latin American settings. We present the case of a 72-year-old female patient with RPGN in the context of positive PR3-ANCA serology and an IgG kappa monoclonal component, compatible with MGUS.

## 2. Case Presentation

A 72-year-old woman with a history of arterial hypertension, type 2 diabetes mellitus, and hypothyroidism, treated with enalapril, amlodipine, empagliflozin/metformin, and levothyroxine, presented with a one-day history of somnolence, disorientation, and markedly elevated blood pressure. Initial laboratory tests revealed acute kidney injury (AKI), with a serum creatinine level of 486.21 µmol/L and a blood urea nitrogen level of 22.38 mmol/L, compared with a prior baseline creatinine of 70.72 µmol/L three months earlier. This corresponded to an estimated glomerular filtration rate (eGFR) of 78 mL/min/1.73 m^2^, calculated using the 2021 CKD-EPI creatinine equation. Hyperkalemia, hyperphosphatemia, hyponatremia, and metabolic acidosis were also documented, and urgent hemodialysis was initiated. Following the initial dialysis sessions, the patient’s acid–base status improved and serum potassium levels decreased to below 5.0 mmol/L. During hospitalization, hyperphosphatemia persisted despite treatment with phosphate binders, including aluminum hydroxide, calcium carbonate, and sevelamer, whereas serum sodium levels fluctuated between mild hyponatremia and values within the normal range. Hemodynamically, the patient was initially unstable in the context of hypertensive emergency and volume overload but stabilized after dialysis, maintaining acceptable blood pressure levels during most of the hospital stay.

At admission, initial laboratory evaluation revealed leukocytosis (14.24 × 10^3^/µL) with neutrophil predominance, without clinical, microbiological, or other paraclinical findings clearly suggestive of an active infection at that time. A 24 h urine collection showed proteinuria of 602 mg, consistent with a subnephrotic range, with a reduced urine volume (750 mL). Urinalysis revealed an active urinary sediment, with proteinuria, significant hematuria (250 erythrocytes/µL), eumorphic erythrocytes (6–8 per high-power field), and mild leukocyturia (25 leukocytes/µL). These findings, together with the rapid deterioration of renal function, were suggestive of RPGN, prompting a comprehensive etiologic workup that included testing for chronic infections, an endocrine profile showing elevated TSH—which prompted an increase in levothyroxine from 100 to 125 µg daily—and elevated parathyroid hormone (PTH) attributable to disordered mineral metabolism in the context of AKI, as well as evaluation for autoimmunity and neoplasms. Concerning the autoimmune workup, indirect immunofluorescence (IIF) for ANCA was negative, but specific PR3-ANCA positivity was detected by ELISA (120 U/mL) (Table 1).

Contrast-enhanced computed tomography scans of the chest, abdomen, and pelvis were performed to exclude an underlying solid malignancy as a potential paraneoplastic trigger and to assess possible extra-renal involvement. At the time imaging was obtained, the patient had already initiated hemodialysis; therefore, the potential diagnostic benefit was considered to outweigh the risk associated with contrast exposure in this critically ill setting. No masses or lymphadenopathy suggestive of a solid malignancy were identified.

In the setting of rapidly progressive renal failure, normocytic anemia, and disturbances of bone–mineral metabolism, serum protein electrophoresis and immunofixation were performed to rule out a monoclonal gammopathy, particularly an MGRS, as a possible underlying etiology. Electrophoresis showed a monoclonal spike in the gamma region, which was confirmed by immunofixation as an IgG with a kappa light chain. Quantitative measurement of the monoclonal component (M-protein) was not available, as densitometric quantification is not routinely performed in our institutional laboratory due to technical and resource limitations. Evaluation for lymphoproliferative disease included a search for osteolytic lesions by plain radiographs of the skull, spine, and long bones, without pathological findings. Likewise, Congo red staining of subcutaneous adipose tissue was negative for amyloid deposits. To rule out multiple myeloma, a bone marrow biopsy was performed, which showed preserved marrow cellularity with a small plasma cell population (<10%), without dysplasia, clonal aggregates, or findings suggestive of malignant hematologic disease. These findings allowed classification of the disorder as MGUS.

**Figure 1:**
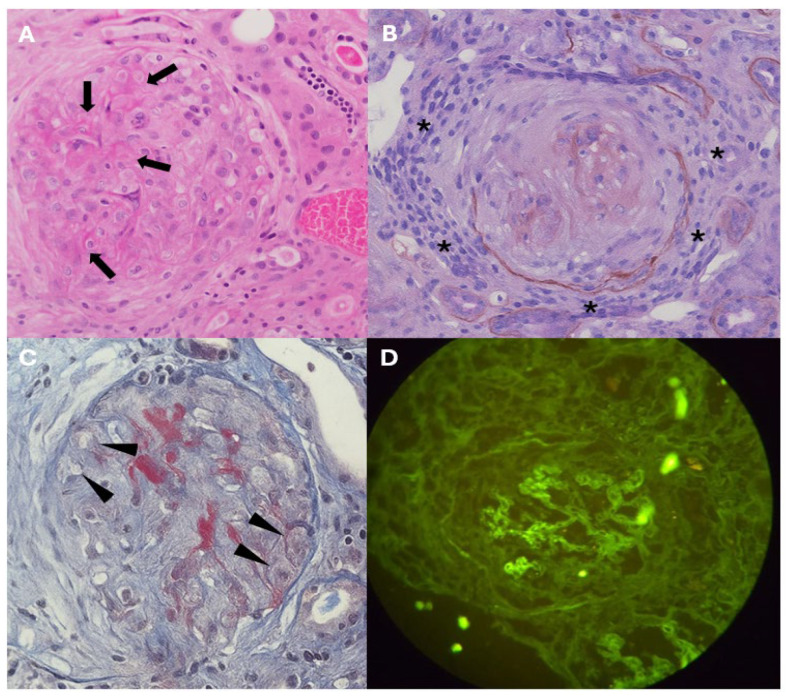
Renal biopsy micrographs in PR3-ANCA-associated pauci-immune necrotizing crescentic glomerulonephritis. (**A**) Glomerulus stained with hematoxylin and eosin (H&E, ×40) showing a central area of fibrinoid necrosis (black arrows) associated with an active extracapillary proliferative lesion characterized by a cellular crescent. (**B**) Jones methenamine silver stain (×40) highlighting the extracapillary cellular crescent (asterisks) and distortion of the glomerular architecture secondary to the necrotizing lesion. (**C**) Masson trichrome stain (×40) demonstrating fine cytoplasmic vacuolization of tubular epithelial cells (arrowheads), consistent with superimposed acute tubular injury. (**D**) Direct immunofluorescence for IgG (×40) showing weak (1+) linear staining along the glomerular basement membranes; although reminiscent of an anti-glomerular basement membrane pattern, the low intensity was considered nonspecific and insufficient to support anti-GBM disease.

A percutaneous renal biopsy was performed, yielding 17 glomeruli for histological analysis. Of these, 29% showed global sclerosis, whereas 52% exhibited active extracapillary proliferative lesions characterized by cellular and fibrocellular crescents. Focal fibrinoid necrosis of the glomerular tuft, a characteristic finding of active vasculitis, was identified in 24% of the glomeruli (Figure 1). The tubulointerstitial compartment showed mild fibrosis and tubular atrophy, estimated to involve approximately 20% of the evaluated parenchyma. Direct immunofluorescence demonstrated faint linear IgG (1+) deposition along the glomerular basement membrane, without light-chain restriction, a pattern considered nonspecific and of insufficient intensity to support anti-glomerular basement membrane (anti-GBM) disease. Electron microscopy revealed moderate thickening of the glomerular basement membranes (400–600 nm) with mesangial matrix expansion and marked podocyte injury, including diffuse foot process effacement, villous transformation, hypertrophy, and focal detachment. No organized electron-dense deposits suggestive of immune complex-mediated disease, amyloidosis, or fibrillary glomerulopathy were identified. Overall, the ultrastructural findings were interpreted as consistent with background diabetic nephropathy. The podocyte alterations were considered most likely secondary to the severe inflammatory and hemodynamic injury associated with the underlying vasculitic process rather than representing a primary podocytopathy. In view of the specific PR3-ANCA positivity and the clinical context of pauci-immune necrotizing glomerulonephritis, ANCA-associated vasculitis was considered more likely.

Immunosuppressive therapy was initiated with intravenous methylprednisolone at a dose of 500 mg daily for three consecutive days, followed by oral prednisolone 50 mg daily. Empiric antibiotic therapy was not initiated at admission because there were no clinical, microbiological, or other paraclinical findings clearly suggestive of active infection at that time, and the overall presentation was considered more consistent with rapidly progressive glomerulonephritis of likely autoimmune origin. However, an occult or subclinical infection cannot be completely excluded. The use of cyclophosphamide as an induction agent was considered; however, its administration was deferred because of the early onset of severe infectious complications. During the hospital course, the patient developed catheter-associated hemodialysis bacteremia, with blood cultures positive for methicillin-sensitive Staphylococcus aureus (MSSA), accompanied by clinical findings compatible with septic emboli. Inflammatory markers initially showed partial improvement; however, at the onset of MSSA bacteremia, a new inflammatory surge was observed, with CRP increasing to 160 mg/L and leukocytes rising to 22.78 × 10^3^/µL with marked neutrophilia. The vascular access was immediately removed, and the antibiotic regimen was adjusted based on susceptibility testing. Cefazolin was initiated at a dose of 1 g intravenously every 24 h, administered after hemodialysis on dialysis days.

**Figure 2:**
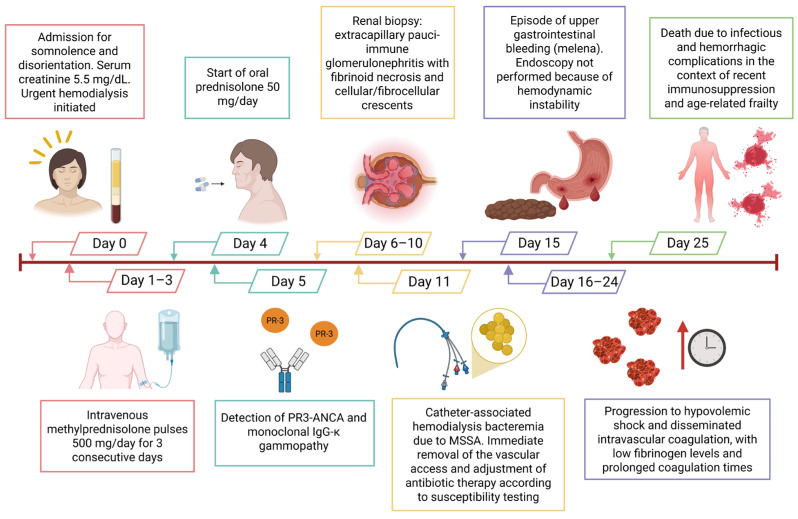
Timeline of the patient’s clinical course and management. Abbreviations: Proteinase 3–antineutrophil cytoplasmic antibodies (PR3-ANCA). Immunoglobulin G (IgG), Monoclonal gammopathy of undetermined significance (MGUS), Methicillin-susceptible Staphylococcus aureus (MSSA).

During hospitalization, the patient underwent seven hemodialysis sessions; however, the last sessions were limited due to episodes of intradialytic hypotension. Standard intradialytic heparin anticoagulation was used during the early hemodialysis sessions to prevent extracorporeal circuit clotting; however, it had been administered before the onset of upper gastrointestinal bleeding. She subsequently developed upper gastrointestinal bleeding, suspected based on melena; however, endoscopy was not performed because of hemodynamic instability. The patient progressed to hypovolemic shock and disseminated intravascular coagulation (DIC), evidenced by prolonged coagulation times and decreased fibrinogen. She died on day 25, attributable to infectious and hemorrhagic complications in the context of recent immunosuppression and frailty associated with advanced age (Figure 2).

## 3. Discussion

ANCA-associated vasculitis with PR3 specificity is a rare systemic inflammatory disease, characterized by necrosis of small- and medium-sized vessels, predominantly glomerular, and is classically associated with granulomatosis with polyangiitis (GPA) [11,12]. Its incidence varies by ethnic and geographic background; for instance, in Europe and North America, rates of 5–16 cases per million inhabitants per year have been reported [13]. The only available population-based study comes from Argentina, where, in a cohort of more than 349,000 individuals, an incidence of 9 cases per million person-years and a prevalence of 7.4 per 100,000 inhabitants for GPA was reported [14,15]. To date, no further epidemiological studies have been published in Latin America, and regional information therefore remains limited.

This condition typically occurs in middle-aged or older adults, with a similar distribution between sexes, although some reports show a slight female predominance [16]. Initial symptoms are usually constitutional—fever, asthenia, weight loss—and are accompanied by myalgias or arthralgias. Upper airway involvement (chronic sinusitis, otitis media, nasal lesions) is an early and characteristic manifestation of the disease [17]. Renal involvement occurs in approximately 50–75% of cases and usually presents as necrotizing pauci-immune glomerulonephritis. It is characterized by dysmorphic hematuria, red blood cell casts, and typically subnephrotic proteinuria, with rapid progression if early intervention is not undertaken [18].

Diagnosis is based on the integration of clinical, serological, and histopathological findings. ANCA detection combines IIF, which is more sensitive but less specific, with second-generation assays such as ELISA or bead-based techniques, which provide higher specificity for PR3 (proteinase 3) and MPO (myeloperoxidase). Overall, approximately 80–90% of patients with GPA or microscopic polyangiitis (MPA) are ANCA-positive; GPA is predominantly associated with PR3-ANCA (60–75%), whereas MPA is associated with MPO-ANCA (55–65%) [2].

Concordance between IIF and immunoassay is usually high in primary vasculitides; however, when discordance occurs, the possibility of secondary disease, drug-induced forms, or early stages with a low immunologic burden should be considered. Technical factors may also play a role, such as low serum autoantibody concentrations, recognition of epitopes not detected by IIF, or methodological variability among laboratories. For these reasons, serological results should always be interpreted within the clinical context, and in the setting of persistent suspicion, the diagnosis must be confirmed by biopsy and clinicopathologic correlation [19,20].

Differential diagnoses included kidney-limited vasculitis (more frequent in MPO-ANCA) and double-positive ANCA/anti-GBM syndrome, although the latter could not be fully excluded serologically because of reagent unavailability. Drug-induced vasculitis, systemic infections, malignancies, and other AAV mimics were also considered [21,22]. All of these diagnoses were reasonably excluded based on the clinical, serological, and pathological findings in our case. The absence of lower respiratory tract symptoms, alveolar hemorrhage, or extrarenal involvement and imaging without findings suggestive of malignancy supported the diagnosis of PR3-ANCA-mediated pauci-immune necrotizing glomerulonephritis, with no evidence of secondary causes [23].

Renal biopsy was essential to define the etiology of kidney injury in the setting of a detected IgG kappa monoclonal protein, where the main challenge was to distinguish between ANCA vasculitis and MGRS [24]. The principal reason is that the presence of a monoclonal protein does not necessarily imply that the gammopathy causes renal damage. In cohort studies, up to 40–45% of patients with monoclonal gammopathy and renal injury show MGRS lesions on biopsy, whereas the remainder have lesions unrelated to monoclonality, such as nephrosclerosis, diabetic nephropathy, or vasculitis [24,25,26]. In ANCA vasculitis, the typical lesion is extracapillary necrotizing pauci-immune glomerulonephritis, with minimal or absent immunostaining. In contrast, MGRS is characterized by organized or non-organized monoclonal deposits in glomeruli or tubules, with light-chain restriction and, in some subtypes, Congo red positivity. Thus, biopsy allows determination of whether renal involvement is due to vasculitis, MGRS, or their coexistence [27]. In our case, histological findings were conclusive: cellular and fibrocellular crescents, fibrinoid necrosis, and absence of structured deposits. Immunofluorescence showed weak linear IgG (1+) staining without light-chain restriction or a monoclonal pattern. In the presence of strong PR3-ANCA positivity, the final diagnosis was pauci-immune glomerulonephritis due to PR3-ANCA-associated vasculitis, ruling out gammopathy-mediated renal involvement.

The coexistence of monoclonal gammopathy and ANCA-related renal disease should be interpreted cautiously. Although published case reports of biopsy-proven MGUS coexisting with ANCA-associated glomerulonephritis remain limited (Table 2) [6,7,8,9,28], available evidence suggests that this overlap may be underrecognized rather than unequivocally exceptional. Early work demonstrated ANCA reactivity in some sera from patients with monoclonal gammopathies, although classical vasculitic manifestations were not observed, indicating that serological overlap alone does not establish pathogenic ANCA-associated vasculitis [29]. More recent cohorts have further suggested that monoclonal gammopathy may be more frequent in pauci-immune or ANCA-related renal disease than previously appreciated [10]. In this context, the value of our case lies not in claiming absolute rarity, but in documenting a biopsy-resolved diagnostic overlap—PR3-ANCA-positive pauci-immune necrotizing crescentic glomerulonephritis with coexisting IgGκ MGUS—in a Latin American patient, in whom distinguishing ANCA-associated glomerulonephritis from MGRS had direct therapeutic implications. Among the previously published cases, most patients were Japanese men aged 46 to 76 years and had MPO-ANCA positivity, with only one prior PR3-ANCA case. Paraproteins were predominantly IgG- or lambda-related, and kidney biopsy generally excluded overt MGRS by showing no organized deposits or light-chain restrictions. In contrast, our patient was an older Latin American woman with strong PR3-ANCA positivity and a fulminant renal presentation, further expanding the clinical and geographic spectrum of this diagnostically challenging overlap (Table 2).

In the published cases, treatment included glucocorticoids and cyclophosphamide, with or without plasmapheresis/rituximab, and most patients evolved favorably without serious complications. This strategy is supported by the KDIGO 2024 and EULAR 2022 guidelines, which recommend, as an induction regimen, the combination of steroids with rituximab or cyclophosphamide in patients with severe renal involvement, adjusting treatment intensity according to age, comorbidities, and infectious risk [30,31,32].

In contrast to what has been reported in the literature, our patient had a fulminant course that required immediate initiation of hemodialysis but precluded completion of the immunosuppressive regimen due to early infectious and hemorrhagic complications. This fatal outcome, likely favored by advanced age, clinical frailty, and rapid disease progression, highlights the vulnerability of specific subgroups—such as older adults with comorbidities—to the inherent risks of intensive immunosuppression. It also underscores the need for personalized strategies in high-risk settings.

Distinguishing between MGRS and ANCA vasculitis is critical because treatment is radically different: MGRS requires therapy directed at the immunoglobulin-secreting clone, whereas ANCA vasculitis is treated with conventional immunosuppression [33]. Induction therapy aims to control inflammation and preserve organ function rapidly. In cases with severe organ involvement, such as rapidly progressive renal compromise, the KDIGO 2024 and EULAR 2022 guidelines recommend initiating intravenous methylprednisolone pulses (500–1000 mg/day for 3 to 5 days), followed by oral prednisone with accelerated tapering, a strategy supported by recent clinical trials showing that rapid glucocorticoid reduction decreases the risk of infections without compromising efficacy in remission induction [31,32,34]. As immunosuppressive agents, intravenous pulse cyclophosphamide and rituximab have demonstrated equivalent efficacy [35,36]. Plasmapheresis, following the PEXIVAS trial, is not routinely recommended, although it may be considered in severe alveolar hemorrhage or rapidly progressive renal disease, with still controversial evidence [34]. The induction phase generally lasts 3 to 6 months, depending on clinical response and functional recovery.

In this case, recommended management was initiated with methylprednisolone pulses and oral prednisone; however, induction with cyclophosphamide could not be performed due to *Staphylococcus aureus* bacteremia and, subsequently, upper gastrointestinal bleeding complicated by disseminated intravascular coagulation. These conditions contraindicated initiation of cytotoxic immunosuppression and forced discontinuation of the complete induction regimen, which likely impaired disease control and contributed decisively to the fatal outcome.

Average survival varies with renal function, exceeding 20 years in patients with preserved renal function but decreasing to around 12 years in those with significant renal impairment [37]. These findings have been corroborated in older adults (>65 years), in whom advanced age and renal dysfunction at diagnosis are recognized as the main predictors of mortality [38].

This case illustrates a clinically important and likely underrecognized overlap between PR3-ANCA vasculitis and MGUS. It also provides additional evidence from a Latin American population, thereby expanding the reported clinical and geographic spectrum of this diagnostically challenging presentation.

Among the limitations of this report, we acknowledge that although IgG staining was weak (1+), it would have been preferable to complement the evaluation with serum anti-glomerular basement membrane (anti-GBM) antibodies in order to more confidently exclude a possible double-positive syndrome. However, this test could not be performed due to the unavailability of the required reagent at our institution. Congo red staining was not performed on the kidney biopsy specimen because this technique was not routinely available at our institution during the patient’s clinical course. Electron microscopy was performed subsequently and did not show organized deposits suggestive of amyloid or fibrillary glomerulopathy; however, this finding should be interpreted as complementary and not as the reason for omitting Congo red staining. In addition, intravenous immunoglobulin (IVIG) could theoretically have been considered as a salvage therapeutic option in a scenario where both cyclophosphamide and rituximab are contraindicated. Nevertheless, there is currently no robust evidence supporting IVIG as an effective induction therapy for ANCA-associated vasculitis (AAV) with severe renal involvement, and available data regarding its efficacy remain limited; therefore, it is not included in standard induction protocols [39]. Finally, the absence of long-term clinical follow-up further limits the analysis, as the patient experienced a fatal outcome.

Nevertheless, the case retains high clinical and academic value, clearly illustrating the diagnostic and therapeutic challenges posed by the coexistence of monoclonal gammopathy and ANCA vasculitis. It reinforces the need for individualized strategies in older adults, as well as for generating more regional data to enrich the global understanding of these poorly documented associations.

## 4. Conclusions

This case represents a clinically important and likely underrecognized overlap between PR3-ANCA-associated pauci-immune glomerulonephritis and MGUS. Renal biopsy was decisive in distinguishing vasculitic renal injury from paraprotein-related disease and establishing the final diagnosis. Its main contribution lies in illustrating the diagnostic value of kidney biopsy in a real-world Latin American setting, particularly in patients with monoclonal gammopathy in whom MGRS must be carefully excluded.

In addition, it underscores the need to consider vasculitis in patients with paraproteinemia and acute kidney injury, even when initial serology is ambiguous, and reinforces the importance of adapting therapeutic regimens for older adults with clinical frailty in whom the therapeutic window is limited and decisions must be individualized. This report expands knowledge in a sparsely documented field and supports the generation of further evidence in regional settings.

## Figures and Tables

**Table 1:** Laboratory findings in the patient.

Test	Result	Reference Range
HbA1c	6.91%	4.0–5.6%
Serum albumin	3.08 g/dL	3.5–5.0 g/dL
24 h proteinuria	602 mg/24 h	<150 mg/24 h
24 h urine volume	750 mL	800–2000 mL
iPTH	184 pg/mL	10–65 pg/mL
ANA	Negative	Negative
Anti-dsDNA	Negative	Negative
Complement C3	98 mg/dL	90–180 mg/dL
Complement C4	15.8 mg/dL	10–40 mg/dL
Anti-MPO	1.1 U/mL	<5 U/mL
Anti-PR3	120 U/mL	<10 U/mL
c-ANCA or p-ANCA (IIF)	Negative	Negative
IgG	1328 mg/dL	700–1600 mg/dL
IgA	245 mg/dL	70–400 mg/dL
IgM	90.1 mg/dL	40–230 mg/dL
TSH	12.3 µIU/mL	0.4–4.0 µIU/mL
RPR	Non-reactive	Non-reactive
HBsAg	0.307 COI	<0.89 COI
Hepatitis C virus Ab	0.03 COI	<0.9 COI
HIV	Negative	Negative
Urinalysis	Proteins (75 mg/dL), significant hematuria (250 erythrocytes/µL), eumorphic erythrocytes (6–8 per high-power field), and mild leukocyturia (25 leukocytes/µL)	N/A

**Abbreviations:** Glycated hemoglobin (HbA1c), intact parathyroid hormone (iPTH), antinuclear antibodies (ANAs), anti-double-stranded DNA antibodies (anti-dsDNAs), anti-myeloperoxidase antibodies (anti-MPOs), anti-proteinase 3 antibodies (anti-PR3s), immunoglobulin G (IgG), immunoglobulin A (IgA), immunoglobulin M (IgM), thyroid-stimulating hormone (TSH), rapid plasma reagin (RPR), hepatitis B surface antigen (HBsAg), hepatitis C virus antibody (hepatitis C virus Ab), human immunodeficiency virus (HIV).

**Table 2:** Summary and detailed comparison of reported cases of MGUS coexisting with ANCA-associated vasculitis.

Author, Year	Country	Age (Years), Sex	Plasma Cell Dyscrasia (MGUS Subtype)	ANCA Serology	Presenting Symptoms	Renal Involvement (Labs/Biopsy)	Extrarenal Involvement	Initial Treatment	Required Dialysis	Outcome/Follow-Up
Takizawa et al., 2017 [6]	Japan	76, Male	Bence Jones λ (MGUS)	MPO-ANCA (+); PR3-ANCA (–)	Chronic cough, otalgia, nasal discharge, hematuria	Hematuria; necrotizing crescentic GN	Pulmonary cavity, otitis media, and pulmonary nodules	IV methylprednisolone + rituximab, followed by oral prednisone	No	Renal and clinical remission
Hishida et al., 2022 [7]	Japan	72, Male	IgG κ MGUS (elevated κ FLC)	MPO-ANCA (+)	Ocular pain, visual disturbance, renal failure	AKI; TIN with peritubular capillaritis	Optic neuropathy; pulmonary nontuberculous mycobacterial infection	Prednisone (monotherapy)	No	Stable renal function and improvement of ocular symptoms
Ueno et al., 2022 [8]	Japan	68, Male	IgG λ MGUS	PR3-ANCA (+) (20.9 U/mL)	Edema, hematuria, hypertension	Nephritic syndrome; crescentic GN with mesangial immune-complex deposits	None prominent	Prednisolone	No	Renal and serologic remission; MGUS persisted
Li et al., 2019 [28]	China	46, Male	IgG κ MGUS	Dual MPO-ANCA + anti-GBM	Fatigue, anorexia, oliguria, gastrointestinal discomfort	RPGN; crescentic GN with linear IgG deposition (anti-GBM positive)	Systemic, nonspecific (GI symptoms, fatigue)	Plasmapheresis + steroids + IV cyclophosphamide	Yes (HD-dependent)	Chronic dialysis dependence
Tsuji et al., 2023 [9]	Japan	58, Male	λ MGUS	MPO-ANCA (+) (8.28 IU/mL)	Arthralgias, malaise, hematuria, proteinuria	Mild proteinuria/hematuria; pauci-immune GN + TIN	Sjögren syndrome and sarcoidosis (lymphadenopathy, granulomas)	Prednisolone + IV cyclophosphamide	No	Renal recovery, MPO normalization, and multi-disease remission
Current case, 2025	Colombia	72, Female	IgG κ MGUS (serum immunofixation)	PR3-ANCA (+) (120 U/mL)	Uremic encephalopathy, fatigue, dyspnea, anemia, hematuria	RPGN; extracapillary proliferative GN with fibrinoid necrosis and mild linear IgG (+) deposition along the GBM	None prominent	Hemodialysis, steroids (IV pulses + oral); cyclophosphamide could not be initiated	Yes (HD-dependent)	Death on day 25 (DIC + upper gastrointestinal bleeding)

## Data Availability

The data supporting the findings of this study are not publicly available due to patient privacy and ethical restrictions.
